# Complete Genome Sequence of *Streptomyces parvulus* 2297, Integrating Site-Specifically with Actinophage R4

**DOI:** 10.1128/genomeA.00875-16

**Published:** 2016-08-25

**Authors:** Tomoyasu Nishizawa, Takamasa Miura, Chizuko Harada, Yong Guo, Kazuhiko Narisawa, Hiroyuki Ohta, Hideo Takahashi, Makoto Shirai

**Affiliations:** a Department of Bioresource Science, Ibaraki University College of Agriculture, Ibaraki, Japan; b Biological Resource Center, National Institute of Technology and Evaluation, Tokyo, Japan; c Graduate School of Agriculture and Life Sciences, The University of Tokyo, Tokyo, Japan

## Abstract

*Streptomyces parvulus* 2297, which is a host for site-specific recombination according to actinophage R4, is derived from the type strain ATCC 12434. Species of *S. parvulus* are known as producers of polypeptide antibiotic actinomycins and have been considered for industrial applications. We herein report for the first time the complete genome sequence of *S. parvulus* 2297.

## GENOME ANNOUNCEMENT

*Streptomyces parvulus* produces polypeptide antibiotics, which are synthesized by multifunctional enzymes such as polyketide synthases (PKSs) and nonribosomal polyketide synthases (NRPSs) (1–3). *S. parvulus* 2297 was derived from strain ATCC 12434^T^ by a standard mutagenesis technique (4). Strain 2297 has been utilized as the host of a cosmid vector and in the site-specific recombination of actinophage R4 (5–7), and the integration mechanism between strain 2297 and the R4 phage has been investigated (8–10). These site-specific recombination events have been applied to the gene integration system for hetero-bacterial hosts (11, 12). Although the R4 phage genome sequence has been elucidated (13), the principal host genome sequence currently remains unclear. In order to gain an industrial insight into secondary metabolism and genome engineering by site-specific recombination, the genome sequence of strain 2297 was examined by means of a hybrid assembly based on paired-end sequencing and single-molecule real-time sequencing data.

The strain 2297 DNA genome was sequenced using Illumina MiSeq and PacBio RSII (APRO Life Science Institute, Inc., Naruto, Japan). The paired-end reads from MiSeq were trimmed using sickle version 1.200 with default parameters (https://github.com/najoshi/sickle). The hybrid assembly with MiSeq and PacBio RSII data (34,760,398 paired-end and 222,831 single-end reads, and 110,723 long reads, respectively) was performed by SPAdes (v3.5.0 with the option, –careful) (14). Finishing was performed using GenoFinisher software (15) and the BWA-MEM (v0.7.12) algorithm (16). The alignment with telomere sequences was analyzed by BLAST (17) using strain ATCC 12434 (accession numbers AF038454 and AF038455). The genome sequence of strain 2297 was annotated using the NCBI Prokaryotic Genomes Automatic Annotation Pipeline (PGAP), CAZy database with dbCAN HMM v3.0 (18), and antiSMASH (19).

The genome of strain 2297 consisted of a 7,149,446-bp linear chromosome (coverage of 252.8-fold) with 72.8% G+C content and containing 6,287 coding sequences (CDSs), 18 rRNA genes and 65 tRNA genes, and a 617,085-bp linear plasmid (coverage of 339.5-fold) with 71.9% G+C content and containing 427 CDSs. The telomere sequences in strain 2297 were conserved between the position of 1 to 180 bases in the linear chromosome and that of 616,906 to 617,085 bases in the linear plasmid, which possess 89% and 85% identities in opposite terminal ends, respectively. According to the antiSMASH analysis, 21 and 3 gene clusters related to secondary metabolites were predicted in the chromosome and plasmid, respectively. The type II PKS module, type III PKS module, 4 NRPS modules, and 2 PKS-NRPS hybrid modules were identified in these gene clusters. On the other hand, it was presumed that the host strain possessed an excisionase for site-specific excision because there was no gene for the excision of a prophage on the R4 phage genome (13). A gene encoding excisionase was also not identified on the strain 2297 genome, suggesting the potential of an excisionase that has not yet been identified in site-specific excision.

### Accession number(s).

The genome sequence of *S. parvulus* 2297 has been deposited in the DDBJ/EMBL/GenBank database under the accession numbers CP015866 and CP015867.
